# Ambroxol for the treatment of COVID-19 among hospitalized patients: A multicenter retrospective cohort study

**DOI:** 10.3389/fmicb.2022.1013038

**Published:** 2022-10-06

**Authors:** Yun Lu, Qing-qing Yang, Lin Zhuo, Kun Yang, Hao Kou, Su-yu Gao, Wen Hu, Qiao-li Jiang, Wen-jing Li, Dong-fang Wu, Feng Sun, Hong Cheng, Siyan Zhan

**Affiliations:** ^1^Department of Pharmacy, Zhongnan Hospital of Wuhan University, Wuhan, China; ^2^Department of Epidemiology and Biostatistics, School of Public Health, Peking University, Beijing, China; ^3^Research Center of Clinical Epidemiology, Peking University Third Hospital, Beijing, China

**Keywords:** ambroxol, COVID-19, clinical outcomes, effectiveness, SARS-CoV-2

## Abstract

Ambroxol is a commonly used mucolytic agent principally used to treat respiratory diseases, which may have a role as adjunctive therapy for severe acute respiratory syndrome coronavirus-2 (SARS-CoV-2) infection, but there is lack of evidence about its effectiveness on coronavirus disease-2019 (COVID-19) patients. To study the association between ambroxol use and clinical outcomes among hospitalized patients of COVID-19 infection. We conducted a multicenter retrospective cohort study involving 3,111 patients with confirmed SARS-CoV-2 infection from three hospitals in Wuhan from 19 December 2019 to 15 April 2020, and the primary outcome was in-hospital mortality. COVID-19 patients were classified into ambroxol and non-ambroxol groups based on the administration of ambroxol during hospitalization. Two analyses including propensity score matching (PSM) to obtain a 1:1 balanced cohort and logistic regression were used to control for confounding factors. The average age of 3,111 patients was 57.55 ± 14.93 years old, 127 of them died during hospitalization, and 924 of them used ambroxol. Treatment with ambroxol did not have a significant effect on in-hospital mortality of COVID-19 patients when compared with non-ambroxol in PSM model after adjusting for confounders (8.0% vs. 3.5%, adjusted OR, 1.03 [95% CI, 0.54–1.97], *p* = 0.936). Adverse events such as nausea/vomiting, headache, and rash were comparable between the two groups. Our results suggest that the use of ambroxol is not significantly associated with in-hospital mortality in COVID-19 patients, which provides evidence for evaluating the effects of ambroxol on COVID-19 patient outcomes and may be helpful for physicians considering medication alternatives for COVID-19 patients.

## Introduction

The coronavirus disease-2019 (COVID-19) caused by severe acute respiratory syndrome coronavirus-2 (SARS-CoV-2) has become a global pandemic since January 2020 (Hu et al., 2021). The clinical characteristics range from asymptomatic, mild, moderate to severe symptoms with varied multi-organ pathologies and even death, which brings huge medical burden to all of society (Bal et al., 2020). Diverse pharmacological strategies and vaccines have been used to try to combat COVID-19 (Sanders et al., 2020; John et al., 2021; Sun et al., 2021); however, to date, we still urgently need effective treatments for COVID-19.

Antiviral screening in virtual and *in vitro* study recommended several potential therapeutic drugs for treating COVID-19 (Bocci et al., 2020), among which ambroxol is a commonly used drug to treat acute and chronic respiratory diseases associated with abnormal mucus secretion and transport which may be implicated with its capabilities including anti-inflammatory, antioxidant, antiviral, antibacterial activities and mucociliary clearance activities (Cazan et al., 2018). A previous study also revealed that ambroxol showed weak but broad antiviral activity to inhibit influenza virus activation and trigger antiviral factors release with a reduction of respiratory infections (Kido et al., 2004). Ambroxol also plays a vital role in the release of surfactant, which helps prevent the proliferation of the flu virus, prevent alveolar collapse, and maintain alveolar function (Yang et al., 2002).

As the most critical step during COVID-19 infection, the spike (S) protein of SARS-CoV-2 binds to the angiotensin-converting enzyme 2 (ACE2), and enters host cells *via* the action of transmembrane serine protease 2 (TMPRSS2) subsequently (Hatmal et al., 2020; Hoffmann et al., 2020). A previous study has been reported that ambroxol has a direct impact on the alveolar Type 2 cells (AT2) which highly express ACE2 receptors (Malerba and Ragnoli, 2008). A recent study has demonstrated that ambroxol has good affinity at the human ACE2 site (Kehinde et al., 2022) as well as inhibits the interaction with S protein’s receptor binding (Olaleye et al., 2020), and exhibits inhibitory activity on TMPRSS2, which is one of the key proteases for COVID-19 viral fusion into host cells (Chikhale et al., 2020). Subsequent study has supported that ambroxol could be a SARS-CoV-2 cell entry inhibitor for COVID-19 intervention (Carpinteiro et al., 2021). Owing to the anti-inflammatory effects and an antiviral function as well as a unique stimulatory action on the secretion of surfactant by AT2 cells, research also suggested that repositioning of ambroxol is necessary which could be a promising drug against SARS-CoV-2 infection (Alkotaji, 2020).

Inspired by the above studies suggesting that ambroxol may have an inhibitory effect on SARS-CoV-2, it may be hypothesized that ambroxol may be useful in improving COVID-19 patient outcomes, so this retrospective study was conducted to explore the effects of ambroxol on COVID-19 patient outcomes.

## Materials and methods

### Study design and population

This retrospective cohort study with participants from three hospitals in Wuhan, China was approved by the Medical Ethics Committee of Zhongnan Hospital of Wuhan University (Approval Code 2020098 K), and written informed consent for participation was not required.

**Figure 1:**
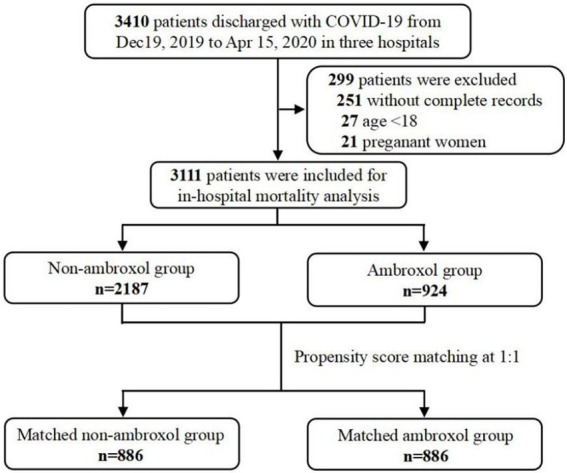
Sampling strategy of COVID-19 admissions in multicenter. COVID-19, coronavirus disease-2019.

Data of hospitalized patients were collected from the electronic medical records system. From 19 December 2019 to 15 April 2020, 3,410 patients diagnosed with COVID-19 based on the guideline for the diagnosis of COVID-19 (Jin et al., 2020) were enrolled. Among these patients, we excluded: (a) 251 patients without key information (such as initial symptoms, demographic characteristics, comorbidities, etc.), (b) 27 patients under the age of 18 years, and (c) 21 pregnant women. As a result, there were 3,111 patients included for in-hospital mortality analysis (Figure 1). The patients’ baseline conditions were classified into mild, moderate, severe, and critical severe cases according to the updated guidelines of diagnosis and treatment of COVID-19 (six version). Then, we defined non-severe group as a collection of mild cases or moderate cases, and defined severe group as a collection of severe cases or critically severe cases.

### Assessment of clinical outcomes and treatment

According to whether ambroxol was used during hospitalization, patients with COVID-19 were classified into non-ambroxol and ambroxol groups. The primary clinical outcome in our analysis was in-hospital mortality of hospitalized COVID-19 patients. The secondary outcomes were the development of adverse events during hospital stay after the ambroxol prescription including headache, rash, and nausea/vomiting.

### Covariates

The following covariates were considered: demographic characteristics (age and gender), baseline clinical characteristics such as clinical status at admission (non-severe group and severe group), symptoms at admission (abnormal chest CT, fever, cough, dyspnea, yes or no), and comorbidities (hypertension, diabetes and chronic kidney disease, yes or no). In addition, other main therapeutic drugs (antiviral, antibiotic, glucocorticoids, Chinese medicine, general nutrients, and immunosuppressor) used were also considered.

### Statistical analysis

Categorical variables in our study were described as frequency (percentages), and continuous variables as mean ± standard deviation (SD) or median ± interquartile range (IQR). For categorical variables, χ2 or Fisher’s exact test was used to make comparisons of proportions in different subgroups; The *t*-tests or Mann–Whitney *U*-test was used for comparisons in continuous variables. Propensity score methods could reduce bias in treatment effects evaluation (Haukoos and Lewis, 2015). In our study, propensity score matching (PSM) was used to decrease the likelihood of confounding in evaluating ambroxol effects.

To examine the association between ambroxol and in-hospital mortality, patients who did and did not use ambroxol were matched 1:1 based on similar or identical propensity scores. This PSM was achieved by *MatchIt* package in R using greedy nearest neighbor matching (maximum caliper distance = 0.1). In our analysis, variables about demographic characteristics, baseline clinical characteristics such as severity and symptoms at admission, and comorbidities were taken in multi-variable logistic regression to calculate propensity scores; equivalence between two groups was examined by the above described methods of categorical and continuous variables. Then, conditional logistic regression with or without drugs adjusted was used to explore associations between ambroxol and outcomes. Furthermore, logistic regression model was also performed to compare with propensity score methods. In the logistic regression, strategies of covariates adjusted were similar to those used in PSM. For safety events, only the number of patients on each group was given. All statistical analyses were performed in R software (version 4.0.2). Statistically significant was considered by a two-tailed *p* < 0.05.

## Results

A total of 3,111 COVID-19 patients were included for the analyses of in-hospital mortality in the cohort, 924 (29.7%) patients received ambroxol treatment throughout the hospital stay (Figure 1). Supplementary information about administrations and dosage appears in Supplementary Tables S1, S2. Among all patients, the median duration of ambroxol treatment was 11 days (IQR [6, 18]). The median doses of ambroxol were 90 mg/day (IQR [60, 180]). The median time interval from admission to initiation of ambroxol prescription was 2 days (IQR [0, 9]; Supplementary Table S1).

Among 3,111 patients, compared with non-ambroxol group, patients who received ambroxol were older (56.10 ± 14.90 vs. 60.98 ± 14.42, *p* < 0.001) and showed more severe condition at admission (9.6% vs. 23.5%, *p* < 0.001). Compared with non-ambroxol group, ambroxol users were more likely to have abnormal chest CT (78.6% vs. 82.0%, *p* = 0.031), cough (54.5% vs. 61.1%, *p* = 0.001), dyspnea (17.1% vs. 23.8%, *p* < 0.001) at admission, and hypertension (27.1% vs. 35.1%, *p* < 0.001). We found that the antiviral (60.6% vs. 69%, *p* < 0.001), antibiotic (40.3% vs. 65.5%, *p* < 0.001), glucocorticoids (12.9% vs. 28.8%, *p* < 0.001), general nutrients (3.7% vs. 14.5%, *p* < 0.001), and immunosuppressor (5.4% vs. 13.3%, *p* < 0.001) were more frequently used on ambroxol group patients, but the Chinese medicine was more frequently used on non-ambroxol group (76.6% vs. 69.5%, *p* < 0.001; Table 1).

**Table 1:** Demographic and baseline clinical characteristics of 3,111 COVID-19 patients.

Characteristic	Overall (*n* = 3,111)	Non-ambroxol (*n* = 2,187)	Ambroxol (*n* = 924)	Value of *p*
**Demographic characteristics**				
Age [mean (SD)]	57.55 (14.93)	56.10 (14.90)	60.98 (14.42)	**<0.001**
Female (%)	1,644 (52.8)	1,182 (54.0)	462 (50.0)	**0.043**
**Baseline clinical characteristics, *n* (%)**				
**Severity at admission, *n* (%)**				
Severe group	426 (13.7)	209 (9.6)	217 (23.5)	**<0.001**
**Symptoms at admission, *n* (%)**				
Abnormal chest CT	2,476 (79.6)	1718 (78.6)	758 (82.0)	**0.031**
Fever	1,660 (53.4)	1,158 (52.9)	502 (54.3)	0.506
Cough	1756 (56.4)	1,191 (54.5)	565 (61.1)	**0.001**
Dyspnea	595 (19.1)	375 (17.1)	220 (23.8)	**<0.001**
**Comorbidities, *n* (%)**				
Hypertension	917 (29.5)	593 (27.1)	324 (35.1)	**<0.001**
Diabetes	374 (12.0)	260 (11.9)	114 (12.3)	0.77
Chronic kidney disease	90 (2.9)	57 (2.6)	33 (3.6)	0.177
**Medications, *n* (%)**				
Antiviral	1964 (63.1)	1,326 (60.6)	638 (69.0)	**<0.001**
Antibiotic	1,487 (47.8)	882 (40.3)	605 (65.5)	**<0.001**
Glucocorticoids	549 (17.6)	283 (12.9)	266 (28.8)	**<0.001**
Chinese medicine	2,317 (74.5)	1,675 (76.6)	642 (69.5)	**<0.001**
General nutrients	215 (6.9)	81 (3.7)	134 (14.5)	**<0.001**
Immunosuppressor	241 (7.7)	118 (5.4)	123 (13.3)	**<0.001**

Bold values indicated *p* < 0.05.

### Primary outcomes

Eight hundred and eighty-six participants from the ambroxol group were 1:1 matched with non-ambroxol group. The demographic characteristics and baseline clinical characteristics were well-balanced in two groups (Supplementary Table S3). In the unadjusted analysis, ambroxol use had a significant association with an increased in-hospital mortality in COVID-19 patients (OR, 4.99 [95% CI, 3.44–7.32], *p* < 0.001). In PSM analysis, after adjusting age, sex, severity and symptoms at admission, comorbidities, and medications, ambroxol was not significantly associated with in-hospital mortality (adjusted OR, 1.03 [95% CI, 0.54–1.97], *p* = 0.936). Similarly, the use of ambroxol was not significantly associated with in-hospital mortality in patients with COVID-19 in logistic model after adjusting age, sex, severity and symptoms at admission, comorbidities and medications (adjusted OR, 1.55 [95% CI, 0.99–2.43], *p* = 0.053; Table 2).

**Table 2:** Associations between inpatient ambroxol use and in-hospital mortality.

Analysis	In-hospital mortality	
	OR (95%CI)	Value of *p*
Unadjusted	4.99 (3.44, 7.32)	<0.001
**PSM (1:1 matching)**		
With matching^†^	2.33 (1.52, 3.58)	<0.001
With matching and further adjustment for medications^‡^	1.03 (0.54, 1.97)	0.936
**Logistic regression model (LRM)**		
Adjustment for age, sex, severity, symptoms, and comorbidities	3.07 (2.06, 4.62)	<0.001
Adjustment for age, sex, severity, symptoms, comorbidities, and medications	1.55 (0.99, 2.43)	0.053

OR, odd ratio; CI, confidence interval; PSM, propensity score matching.

† Ambroxol and non-ambroxol groups were matched by age, sex, severity and symptoms at admission, and comorbidities in propensity score matching.

‡ Ambroxol and non-ambroxol groups were matched by age, sex, severity and symptoms at admission, and comorbidities in propensity score matching, and conditional logistic regression models were additionally adjusted for medications.

**Figure 2:**
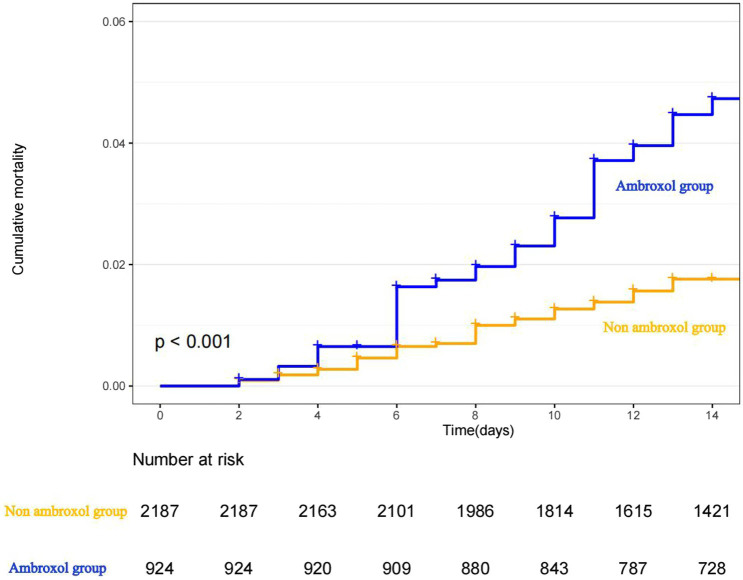
Cumulative mortality curves of in-hospital mortality.

The Kaplan–Meier curve for cumulative probability of in-hospital mortality by ambroxol use is shown in Figure 2. Follow-up started from the admission and continued for 14 days or until death when applicable for each patient.

The effects of ambroxol on 426 severe patients were also presented. Among them, compared with non-ambroxol group, severe patients who received ambroxol were older (61.80 ± 14.89 vs. 66.73 ± 13.55, *p* < 0.001). Compared with non-ambroxol group, severe ambroxol users were more likely to have dyspnea (26.3% vs. 40.6%, *p* = 0.003) at admission and chronic kidney disease (3.3% vs. 9.2%, *p* = 0.022). We found that the antibiotic (48.3% vs. 73.7%, *p* < 0.001), glucocorticoids (15.3% vs. 36.9%, *p* < 0.001), general nutrients (9.1% vs. 33.2%, *p* < 0.001), and immunosuppressor (8.1% vs. 16.1%, *p* = 0.018) were more frequently used on ambroxol group, but the Chinese medicine was more frequently used on non-ambroxol group (70.3% vs. 60.4%, *p* = 0.04; Supplementary Table S4).

One hundred and sixty-nine severe patients from the ambroxol group were 1:1 matched with non-ambroxol group. The two groups were well-balanced in demographic characteristics and baseline clinical characteristics (Supplementary Table S5). In the unadjusted analysis, ambroxol use had a significant association with increased in-hospital mortality in severe COVID-19 patients (OR, 3.29 [95% CI, 1.86–6.09], *p* < 0.001). In PSM analysis, after adjusting age, sex, symptoms at admission, comorbidities, and medications, ambroxol was not associated with significance in in-hospital mortality (adjusted OR, 1.39 [95% CI, 0.62–3.14], *p* = 0.421). Similarly, the use of ambroxol was not associated with in-hospital mortality in severe patients with COVID-19 in logistic model after adjusting age, sex, symptoms at admission, comorbidities, and medications (adjusted OR, 1.26 [95% CI, 0.63–2.54], *p* = 0.520; Supplementary Table S6).

### Secondary outcomes

Patients who received ambroxol had comparable incidences of nausea/vomiting, headache, and rash in comparison with those who did not receive ambroxol (Table 3).

**Table 3:** Safety outcomes and adverse events.

Outcomes	Unmatched			Propensity score matching^†^		
	Non-ambroxol (*n* = 2,187)	Ambroxol (*n* = 924)	Value of *p*	Non-ambroxol (*n* = 886)	Ambroxol (*n* = 886)	Value of *p*
Nausea/vomiting (*n*, %)	11 (0.5)	6 (0.6)	0.810	5 (0.6)	6 (0.7)	> 0.999
Headache (*n*, %)	9 (0.4)	2 (0.2)	0.612	4 (0.5)	2 (0.2)	0.687
Rash (*n*, %)	7 (0.3)	7 (0.8)	0.170	6 (0.7)	7 (0.8)	> 0.999

^†^Ambroxol and non-ambroxol groups were matched by demographic characteristics and baseline clinical characteristics in propensity score matching.

## Discussion

This is the first multi-center retrospective study focused on the use of ambroxol in COVID-19 patients in real-world practice to our knowledge. Our study is the unique registered clinical trial on the safety and effectiveness of ambroxol in COVID-19 patients in China till now (ChiCTR2000038160). In comparison with prior studies on therapeutic drugs for COVID-19 treatment, our study has involved a relatively larger sample size that ensured sufficient statistical power even for subgroup analysis. In this study, we did not observe the benefit of ambroxol on the in-hospital mortality in COVID-19 patients.

The incidence of adverse effects after the ambroxol prescription was analyzed which revealed no significant difference between ambroxol and non-ambroxol groups. Our work first indicated that the application of ambroxol in COVID-19 patients is clinical and statistic safe, and did not cause any intolerable or severe side reactions.

We found that 29.7% of COVID-19 patients received ambroxol. Compared with non-ambroxol group, patients who received ambroxol were older, which has shown elevated risk of mortality due to SARS-Cov-2 (Liu et al., 2020). Besides, chronic comorbidity such as hypertension was more common in ambroxol group, which is also a clinical risk factor associated with COVID-19 (Dessie and Zewotir, 2021). Furthermore, most of the ambroxol users were severe cases. A notably higher proportion of ambroxol-receiving patients presented with abnormal chest CT, cough, and dyspnea at admission compared with non-ambroxol groups. Consequently, patients who received ambroxol were prescribed with more medications. The antiviral, antibiotic glucocorticoids, general nutrients, and immunosuppressor treatments were more frequently used on ambroxol group patients, which inferred that the condition of these patients was worse indicating a risk of poor prognosis. Prescription of ambroxol was commonly to these individuals and hence strengthening the difference between the non-ambroxol and the ambroxol group. It is possible that these factors may counteract the effectiveness of ambroxol treatment. Consistently, ambroxol treatment on COVID-19 patients did not have a significant impact on the in-hospital mortality.

As the COVID-19 keeps on evolving, alleviating the symptoms of COVID-19 infective patients in virtue of drugs is still very important. Current data suggest that mucolytic agents may contribute to ameliorate COVID-19 lung disease (Kato et al., 2022). Since ambroxol is effective in removing mucus and ameliorating inflammation in the airway (Beeh et al., 2008; Ge et al., 2016; Zhang et al., 2016; Cao et al., 2019) and the safety of ambroxol is widely acceptable, ambroxol may still be selected as an adjuvant drug to improve respiratory symptoms of COVID-19 symptoms. Ambroxol has been regarded as not only an expectorant but also a potent promoter of lung surfactant synthesis (Takano, 2020). Several studies suggested that the aerosolized form of ambroxol with exogenous pulmonary surfactant at the early stages of COVID-19 may provide synergistic benefit, and the inhalation pattern of ambroxol may be better than systemic administration due to the participation of nasal epithelial cells in SARS-CoV-2 infection (Alkotaji, 2020; Kumar, 2020; Carpinteiro et al., 2021). Further clinical trials are required to clarify the discrepancy of dosage form and co-administration of ambroxol.

This study has some unavoidable limitations due to its retrospective observational study design. First, the treatment patterns of COVID-19 patients were varied. Although PSM and logistic models were used to adjust for confounding variables, patients receiving other medical treatments and other unknown confounders have not been fully included in our study and may influence the evaluation of ambroxol. Second, the comorbidities (hypertension, diabetes, and chronic kidney disease, yes or no) were considered instead of comorbidity score in this study since some diseases were not available in the database at admission, including peripheral vascular diseases, myocardial infarction, cerebrovascular diseases, peptic ulcer diseases, congestive heart failure, dementia, rheumatologic diseases, and AIDS/HIV. Third, the mortality was defined as in-hospital death, and the deaths of patients discharged were not collected. Forth, the dosage and administration routes of ambroxol were not identical since we did not intervene in the clinical decision. Therefore, patients were defined as ambroxol group once he/she were administrated with ambroxol regardless of the duration. As the daily dosage and type of ambroxol may affect the results, further clinical trials remain carried out to provide strong evidence of the effect of ambroxol by controlling the baseline of patient such as dosage and timing of drug administration. The results of our study can infer association rather than causation.

In conclusion, our results suggest that the use of ambroxol is not significantly associated with in-hospital mortality in COVID-19 patients, which provides evidence for evaluating the effects of ambroxol on COVID-19 patient outcomes and may be helpful for physicians considering medication alternatives for COVID-19 patients.

## Data availability statement

The original contributions presented in the study are included in the article/Supplementary material, further inquiries can be directed to the corresponding author.

## Ethics statement

The studies involving human participants were reviewed and approved by the Medical Ethics Committee, Zhongnan Hospital of Wuhan University (Approval Code 2020098 K). Written informed consent for participation was not required for this study in accordance with the national legislation and the institutional requirements.

## Author contributions

SZ, HC, and FS contributed to the conception and design of the study, and are responsible for the accuracy and integrity of the data analysis. HC, D-fW, LZ, KY, HK, S-yG, WH, Q-lJ, and W-jL were responsible for the acquisition of data and literature research. YL and Q-qY drafted the manuscript and contributed to the analysis and interpretation of data. All authors contributed to the article and approved the submitted version.

## Funding

This study was supported by Boehringer Ingelheim (China) Investment Co., Ltd. (no. 0018.0513), the National Key Technology R&D Program of China (no.2020YFC0840800), Fundamental Research Funds for the Central Universities (no. 2042020kf1019), and Special Project for Director, China Center for Evidence Based Traditional Chinese Medicine (2020YJSZX-2).

## Conflict of interest

The authors declare that the research was conducted in the absence of any commercial or financial relationships that could be construed as a potential conflict of interest.

## Publisher’s note

All claims expressed in this article are solely those of the authors and do not necessarily represent those of their affiliated organizations, or those of the publisher, the editors and the reviewers. Any product that may be evaluated in this article, or claim that may be made by its manufacturer, is not guaranteed or endorsed by the publisher.
