# Supplementary material for: Ambroxol for the treatment of COVID-19 among hospitalized patients: A multicenter retrospective cohort study
Source: Front Microbiol. 2022 Oct 6;13:1013038. doi: 10.3389/fmicb.2022.1013038 (PMC9582747; doi:10.3389/fmicb.2022.1013038)
Supplement: Supplementary file 1 [file Data_Sheet_1.docx]

# Supplement

# Tables

**Table S1.** **Ambroxol prescription patterns of 3111 COVID-19 patients.**

| **Total daily dose (mg/d)** | **Initial prescription** | **Second prescription** | **Third prescription** |
| --- | --- | --- | --- |
| 0.45 | 0/924(0.00) | 0/408(0.00) | 1/188(0.53) |
| 6 | 1/924(0.11) | 0/408(0.00) | 0/188(0.00) |
| 15 | 24/924(2.6) | 7/408(1.72) | 5/188(2.66) |
| 22.5 | 0/924(0.00) | 1/408(0.25) | 1/188(0.53) |
| 30 | 187/924(20.24) | 56/408(13.73) | 23/188(12.23) |
| 45 | 42/924(4.55) | 25/408(6.13) | 16/188(8.51) |
| 60 | 191/924(20.67) | 87/408(21.32) | 35/188(18.62) |
| 75 | 42/924(4.55) | 15/408(3.68) | 4/188(2.13) |
| 80 | 2/924(0.22) | 2/408(0.49) | 0/188(0.00) |
| 90 | 373/924(40.37) | 92/408(22.55) | 33/188(17.55) |
| 120 | 24/924(2.6) | 6/408(1.47) | 2/188(1.06) |
| 135 | 0/924(0.00) | 1/408(0.25) | 0/188(0.00) |
| 150 | 2/924(0.22) | 0/408(0.00) | 3/188(1.6) |
| 180 | 34/924(3.68) | 13/408(3.19) | 10/188(5.32) |
| 240 | 0/924(0.00) | 0/408(0.00) | 0/188(0.00) |
| 270 | 0/924(0.00) | 1/408(0.25) | 1/188(0.53) |
| 300 | 3/924(0.32) | 7/408(1.72) | 4/188(2.13) |
| 360 | 1/924(0.11) | 1/408(0.25) | 0/188(0.00) |
| 450 | 1/924(0.11) | 0/408(0.00) | 0/188(0.00) |
| 500 | 0/924(0.00) | 1/408(0.25) | 0/188(0.00) |
| 600 | 0/924(0.00) | 1/408(0.25) | 0/188(0.00) |
| 900 | 0/924(0.00) | 0/408(0.00) | 1/188(0.53) |
| 1000 | 0/924(0.00) | 0/408(0.00) | 1/188(0.53) |
| 1125 | 0/924(0.00) | 1/408(0.25) | 0/188(0.00) |
| Unknown | 273/924(29.55) | 134/408(32.84) | 71/188(37.77) |
| **Administration route** | | | |
| Oral | 364/924(39.39) | 171/408(41.91) | 84/188(44.68) |
| Atomize | 90/924(9.74) | 62/408(15.2) | 32/188(17.02) |
| Injection | 502/924(54.33) | 189/408(46.32) | 78/188(41.49) |
| Other | 0/924(0.00) | 0/408(0.00) | 1/188(0.53) |

Data are presented as number / total number of ambroxol use (percentage)

**Table S2. Ambroxol therapy among 3111 COVID-19 patients.**

| **Duration of ambroxol therapy, days** | **Results** |
| --- | --- |
| All patients | 11.0 [6.0, 18.0] |
| Survivors | 11.0 [6.0, 18.0] |
| Non-survivors | 10.0 [5.0, 17.0] |
| **Dose, g/day** | |
| All patients | 90.0 [60.0, 180.0] |
| Survivors | 90.0 [60.0, 180.0] |
| Non-survivors | 90.0 [75.0, 180.0] |
| **Duration between hospital admission and ambroxol initiation, days** | |
| All patients | 2.0 [0.0, 9.0] |
| Survivors | 2.0 [0.0, 10.0] |
| Non-survivors | 2.0 [0.0, 6.0] |

Data are presented as median (interquartile range).

**Table S3. Clinical features of patients with COVID-19 after propensity score matching of in-hospital mortality.**

| **Characteristic** | **Propensity score matching^†^** | | **P value** |
| --- | --- | --- | --- |
|  | **Non-ambroxol**  **(n =886)** | **Ambroxol**  **(n = 886)** |  |
| Demographic characteristics | | | |
| Age (mean (SD)) | 60.25 (14.26) | 60.43 (14.29) | 0.793 |
| Female (%) | 478 (54.0) | 448 (50.6) | 0.168 |
| Baseline clinical characteristics, n (%) | | | |
| Clinical status at admission | | | |
| Severe group | 171 (19.3) | 179 (20.2) | 0.676 |
| Symptoms at admission | | | |
| Abnormal chest CT | 743 (83.9) | 723 (81.6) | 0.232 |
| Fever | 504 (56.9) | 486 (54.9) | 0.416 |
| Cough | 558 (63.0) | 539 (60.8) | 0.379 |
| Dyspnea | 189 (21.3) | 195 (22.0) | 0.773 |
| Comorbidities | | | |
| Hypertension | 286 (32.3) | 303 (34.2) | 0.420 |
| Diabetes | 112 (12.6) | 110 (12.4) | 0.943 |
| Chronic kidney disease | 30 (3.4) | 28 (3.2) | 0.894 |

† Ambroxol and non-ambroxol groups were matched by demographic characteristics and baseline clinical characteristics in propensity score matching.

**Table S4. Demographic and baseline clinical characteristics of 426 COVID-19 severe patients.**

| **Characteristic** | **Overall**  **(n = 426)** | **Non ambroxol**  **(n =209)** | **Inpatient ambroxol**  **(n = 217)** | ***p*-value** |
| --- | --- | --- | --- | --- |
| Demographic characteristics | | | | |
| Age (mean (SD)) | 64.31 (14.42) | 61.80 (14.89) | 66.73 (13.55) | **<0.001** |
| Female (%) | 190 (44.6) | 102 (48.8) | 88 (40.6) | 0.106 |
| Baseline clinical characteristics, n (%) | | | | |
| Symptoms at admission, n (%) | | | | |
| Abnormal chest CT | 349 (81.9) | 170 (81.3) | 179 (82.5) | 0.856 |
| Fever | 206 (48.4) | 97 (46.4) | 109 (50.2) | 0.489 |
| Cough | 247 (58.0) | 120 (57.4) | 127 (58.5) | 0.894 |
| Dyspnea | 143 (33.6) | 55 (26.3) | 88 (40.6) | **0.003** |
| Comorbidities, n (%) | | | | |
| Hypertension | 173 (40.6) | 77 (36.8) | 96 (44.2) | 0.146 |
| Diabetes | 74 (17.4) | 36 (17.2) | 38 (17.5) | >0.999 |
| Chronic kidney disease | 27 ( 6.3) | 7 ( 3.3) | 20 ( 9.2) | **0.022** |
| Medications, n (%) | | | | |
| Antiviral | 280 (65.7) | 138 (66.0) | 142 (65.4) | 0.979 |
| Antibiotic | 261 (61.3) | 101 (48.3) | 160 (73.7) | **<0.001** |
| Glucocorticoids | 112 (26.3) | 32 (15.3) | 80 (36.9) | **<0.001** |
| Chinese medicine | 278 (65.3) | 147 (70.3) | 131 (60.4) | **0.04** |
| General nutrients | 91 (21.4) | 19 ( 9.1) | 72 (33.2) | **<0.001** |
| Immunosuppressor | 52 (12.2) | 17 ( 8.1) | 35 (16.1) | **0.018** |

**Table S5. Clinical features of severe patients with COVID-19 after propensity score matching of in-hospital mortality.**

| **Characteristic** | **Propensity score matching^†^** | | ***p*-value** |
| --- | --- | --- | --- |
|  | **Non ambroxol**  **(n =169)** | **Inpatient ambroxol**  **(n = 169)** |  |
| Demographic characteristics | | | |
| Age (mean (SD)) | 64.34 (14.21) | 65.18 (13.92) | 0.583 |
| Female (%) | 82 (48.5) | 69 (40.8) | 0.189 |
| Baseline clinical characteristics, n (%) | | | |
| Symptoms at admission, n (%) | | | |
| Abnormal chest CT | 136 (80.5) | 138 (81.7) | 0.890 |
| Fever | 80 (47.3) | 90 (53.3) | 0.328 |
| Cough | 96 (56.8) | 98 (58.0) | 0.912 |
| Dyspnea | 53 (31.4) | 57 (33.7) | 0.728 |
| Comorbidities, n (%) | | | |
| Hypertension | 66 (39.1) | 64 (37.9) | 0.911 |
| Diabetes | 31 (18.3) | 29 (17.2) | 0.887 |
| Chronic kidney disease | 7 ( 4.1) | 4 ( 2.4) | 0.540 |

† Ambroxol and non-ambroxol groups were matched by demographic characteristics and baseline clinical characteristics in propensity score matching.

**Table S6. Associations between inpatient ambroxol use and in-hospital mortality among severe patients with COVID-19.**

| **Analysis** | **In- hospital mortality** | |
| --- | --- | --- |
|  | OR (95%CI) | *p-*value |
| **Unadjusted** | 3.29 (1.86, 6.09) | <0.001 |
| **PSM (1:1 matching)** |  |  |
| With matching^†^ | 2.47 (1.35, 4.49) | 0.003 |
| With matching and further adjustment for  medications^‡^ | 1.39 (0.62, 3.14) | 0.421 |
| **Logistic regression model (LRM)** |  |  |
| Adjustment for age, sex, symptoms, comorbidities | 2.54 (1.40, 4.79) | 0.003 |
| Adjustment for age, sex, symptoms, comorbidities, and medications | 1.26 (0.63, 2.54) | 0.520 |

Abbreviations: OR=odd ratio; CI=confidence interval; PSM= propensity score matching.

† Ambroxol and non-ambroxol groups were matched by age, symptoms and comorbidities in propensity score matching.

‡ Ambroxol and non-ambroxol groups were matched by age, symptoms and comorbidities in propensity score matching, and conditional logistic regression models were additionally adjusted for medication.
